# One-year effects of orthokeratology lens wear on objective visual quality and corneal densitometry in adolescents with myopia

**DOI:** 10.1371/journal.pone.0355390

**Published:** 2026-08-12

**Authors:** YueXin Chen, NingNa Zhang, YanYing Zhu, HaiYan Xie, YuKun Liu, Ke Zhang, XiaoChen Xu, Jing Wang

**Affiliations:** 1 Department of Ophthalmology, The Second Affiliated Hospital of Anhui Medical University, Hefei, Anhui, China; 2 Department of Ophthalmology, The Affiliated Chuzhou Hospital of Anhui Medical University, Chuzhou, Anhui, China; 3 Department of Ophthalmology, Wuhu City Eye Hospital, Wuhu, Anhui, China; Save Sight Institute, AUSTRALIA

## Abstract

This study aimed to evaluate changes in objective visual quality, corneal wavefront aberrations, and corneal densitometry (CDens) after 1 year of orthokeratology (OK) lens wear in adolescents with myopia and to explore the associations among these parameters. This prospective study enrolled 40 eyes from 25 adolescents with myopia (mean age, 10.32 ± 1.73 years). Objective visual quality parameters, including the objective scatter index (OSI), Strehl ratio (SR), and modulation transfer function cutoff frequency (MTFcutoff), were assessed using the Optical Quality Analysis System II, while corneal wavefront aberrations and CDens were measured using the Pentacam system at baseline and at 1, 3, 6, and 12 months after OK lens wear. Longitudinal changes were analyzed using repeated-measures analysis of variance or the Friedman test, followed by Bonferroni-adjusted post hoc comparisons when appropriate. Compared with baseline, OSI increased significantly at 3, 6, and 12 months, and SR decreased significantly at 12 months, whereas MTF_cutoff_ did not differ significantly across the five visits. The root mean square values of total aberration, higher-order aberrations, and lower-order aberrations of the anterior cornea and total cornea increased significantly at all follow-up visits, whereas posterior corneal aberrations showed no significant changes. CDens increased mainly in the anterior corneal layer and in the central and paracentral zones of the central layer and full-thickness cornea, whereas posterior-layer CDens decreased at 12 months. At 12 months, posterior-layer CDens showed moderate associations with SR and selected corneal aberration RMS values. One year of OK lens wear was associated with deterioration in selected objective visual quality parameters and sustained changes in corneal aberration patterns, while posterior CDens may provide complementary information for assessing long-term optical quality after OK lens wear.

## 1. Introduction

Myopia has become a global pandemic, with its prevalence steadily increasing in recent decades [1]. Orthokeratology (OK) lenses are rigid contact lenses with a reverse-geometry design that are worn overnight to reshape the cornea and temporarily correct myopic refractive error, thereby improving uncorrected visual acuity during the day [2]. Several studies have investigated the role of OK lenses in myopia correction and have confirmed that OK can slow myopia progression [3–5]. Given the long-term use of OK lenses for myopia control, evaluating their effects on objective visual quality and corneal transparency is important for clinical follow-up.

During OK treatment, the cornea is reshaped from a prolate to a more oblate configuration. This alteration increases corneal surface irregularity and consequently induces an elevation in corneal higher-order aberrations (HOAs) [6], including spherical aberration and coma [7]. Previous studies have demonstrated that corneal wavefront aberrations after OK mainly occur on the anterior corneal surface, with significant changes in spherical aberration, vertical coma, and horizontal coma [8–10]. Furthermore, retinal image quality is also influenced by intraocular scattering. Lu et al. reported that optical quality declined after 3 months of OK lens treatment, with a significant increase in the objective scatter index (OSI) and notable reductions in both modulation transfer function cutoff (MTF_cutoff_) and Strehl ratio (SR) [11]. These optical changes may lead to visual disturbances, and patients have reported symptoms such as ghosting, glare, and distortion in dark environments [12]. Therefore, it is necessary to comprehensively evaluate changes in optical quality after OK lens wear in children with myopia.

The cornea is the primary refractive medium of the eye, and its transparency plays a pivotal role in optical function and visual acuity [13]. Corneal densitometry (CDens) is a biological property of corneal tissue that can objectively and quantitatively reflect corneal transparency and health [14]. CDens can be quantified using Pentacam software [15] and has been widely used to assess corneal transparency in conditions such as keratoconus, corneal dystrophy, infectious keratitis, corneal refractive surgery, and systemic diseases involving the cornea [16]. Although OK has already been widely used in clinical practice for myopia control, most follow-up evaluations mainly focus on refractive outcomes, axial length, and corneal topographic changes. Previous studies have separately examined objective visual quality [11] and CDens [17,18] after OK lens wear; however, longitudinal evidence assessing both outcomes over 1 year remains limited. More importantly, the relationship between long-term changes in CDens and objective visual quality has not been clearly established, particularly in adolescents.

The aim of this study was to investigate the effects of 1 year of OK lens wear on objective visual quality, corneal wavefront aberrations, and CDens in myopic adolescents and to explore the associations among these parameters. These findings may help inform the long-term clinical monitoring of adolescents undergoing OK treatment.

## 2. Methods

### 2.1. Participants

This prospective study was conducted at the Department of Ophthalmology of the Second Affiliated Hospital of Anhui Medical University. Participants were recruited between 1 December 2020 and 30 October 2021 and were followed for 1 year. A total of 25 participants (40 eyes) were enrolled and underwent OK lens treatment, among whom 10 participants had unilateral myopia and 15 had bilateral myopia. Because this was an exploratory prospective study, the sample size was based on the number of eligible participants recruited during the study period. The inclusion criteria were age between 8 and 18 years, spherical refraction from −0.50 to −6.00 diopters (D), cylindrical refraction from 0 to −3.00 D, and best-corrected visual acuity of 0.1 logMAR (logarithm of the minimal angle of resolution) or better in each included eye. Participants with acute or chronic eye diseases, a history of ocular surgery, systemic illnesses, or medication use that could potentially impact vision or myopia development were excluded. The study was approved by the Ethics Committee of the Second Affiliated Hospital of Anhui Medical University (YX2020–080) on 23 November 2020 and adhered to the principles of the Declaration of Helsinki. The clinical registration code was ChiCTR2000040744. Written informed consent was obtained from the participants and their guardians, including permission to use medical records as research data.

### 2.2. Examinations

All participants underwent routine ophthalmological examinations before trial lens wear, and corneal fluorescein staining was used for lens fit assessment after trial lens wear. The examinations included slit-lamp microscopy, corneal topography, objective visual quality assessment, corneal wavefront aberration analysis, and corneal densitometry assessment at baseline and at 1, 3, 6, and 12 months after OK lens wear. All examinations were completed within 2 hours of morning lens removal to ensure a consistent assessment window. Measurements were repeated at least three times for each eye, and the average values were used for subsequent analysis.

### 2.3. Orthokeratology lens

The OK lenses used in this study (Dream Vision, China) were made of Boston XO material, with an optic zone diameter of 6 mm and an oxygen permeability of 100 × 10^−11^ (cm^2^ × mLO^2^)/ (s × mL × mmHg). All lenses had an overall diameter of 10.00 to 11.50 mm, a central thickness of 0.15–0.30 mm, and a radius of curvature of 7.50–9.93 mm. The lens fitting procedure was performed according to the manufacturer’s recommended protocol. Participants and their parents were instructed on proper lens hygiene and care, and participants were advised to wear the lenses for at least 8 hours per night.

### 2.4. Optical quality analysis system

Objective visual quality was assessed with the Optical Quality Analysis System II (OQAS II, Visiometrics SL, Spain). Wavefront aberration measurements may overestimate retinal image quality in eyes with HOAs or marked intraocular scatter [19]. The OQAS II provides information on both intraocular scattering and aberrations, allowing a comprehensive and objective evaluation of visual quality. Previous studies have shown good repeatability of OQAS measurements in adults [20] and children [21]. The measured parameters included OSI, SR, and MTF_cutoff_. OSI refers to the objective scatter index and reflects the level of intraocular forward light scatter. It is defined as the ratio of peripheral to central light energy in a point-spread function image obtained using the double-pass technique. An OSI value of less than 1 is generally considered normal. SR is a ratio of the central peak of the point spread function in an aberrated optical system to that in an ideal aberration-free optical system with the same pupil diameter. SR ranges from 0 to 1, with higher values indicating better optical quality; an SR of 1 represents a perfect diffraction-limited optical system [20]. OQAS-based measurements, including SR, may be influenced by factors such as pupil diameter, intraocular scatter, tear-film stability, and optical aberrations [22]. MTFcutoff represents the cutoff spatial frequency of the modulation transfer function and is expressed in cycles per degree. Lower OSI values and higher MTF_cutoff_ and SR values indicate better optical quality [23]. The participants’ head and eye positions were adjusted to ensure that the pupil was precisely aligned with the center of the visual target on the screen.

### 2.5. Corneal wavefront aberrations and densitometry

Corneal wavefront aberrations and CDens were assessed using an Oculus Pentacam system (Oculus, Wetzlar, Germany) by an experienced technician. The integrated Scheimpflug camera acquires 50 sectional images within 2 seconds. Each image captures 500 measurement points on both the anterior and posterior corneal surfaces, allowing a highly accurate three-dimensional model of the anterior segment to be generated. The system provides Zernike polynomial terms that can be displayed as corneal wavefront aberrations [24]. The magnitude of corneal wavefront aberrations was expressed as root mean square (RMS) values derived from Zernike coefficients. The analyzed aberration parameters included total aberration RMS (TOA RMS), higher-order aberration RMS (HOA RMS), and lower-order aberration RMS (LOA RMS) for the anterior cornea, posterior cornea, and total cornea. Corneal densitometry was automatically divided by the software into three depth layers: the anterior 120 μm (anterior layer), the central layer between the anterior and posterior layers, and the posterior 60 μm (posterior layer). In addition, a total corneal densitometry value representing the full corneal thickness without stratification was also obtained. Densitometry analysis was further performed within four concentric zones centered on the corneal apex (0–2 mm, 2–6 mm, 6–10 mm, and 10–12 mm).

**Fig 1:**
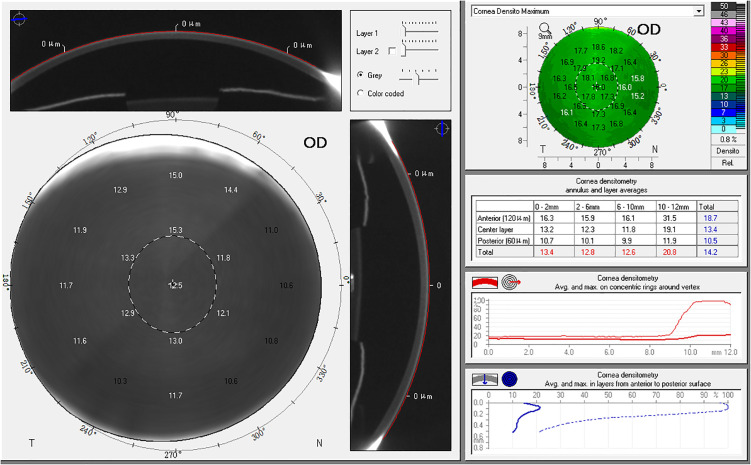
Representative Pentacam image showing corneal densitometry analysis by depth layer and annular zone.

Due to the influence of eyelid condition and measurement error, CDens values within the 10–12 mm zone are less reliable than those in other regions [25]; therefore, this zone was excluded from the analysis. Grayscale units (GSU) indicate corneal opacity on a scale from 0 to 100, with higher values indicating lower transparency and lower values indicating greater transparency [26]. A representative Pentacam image showing the corneal densitometry analysis is provided in Fig 1.

### 2.6. Statistical analysis

Statistical analysis was performed using IBM Corp.’s SPSS 23.0 software (Armonk, NY, USA). The normality of measurement data was assessed using the Kolmogorov-Smirnov test. Differences in MTF_cutoff_, SR, OSI, corneal wavefront aberrations, and CDens across time points before and after OK lens wear were analyzed using repeated-measures analysis of variance when the assumptions for parametric testing were met. When the data were not normally distributed, the Friedman test was used. Significant repeated-measures analysis of variance results were followed by Bonferroni-adjusted pairwise comparisons. Significant Friedman test results were followed by Wilcoxon signed-rank tests with Bonferroni correction. Correlation analysis was performed to evaluate the associations of regional CDens values with objective visual quality parameters (OSI, SR, and MTFcutoff) and corneal wavefront aberration parameters at 12 months after OK lens wear. Pearson correlation analysis was used when both variables followed a normal distribution; otherwise, Spearman correlation analysis was applied. Data are presented as mean ± standard deviation (mean ± SD). P < 0.05 was considered statistically significant.

## 3. Results

A total of 25 participants, including 13 males and 12 females, with a mean age of 10.32  ±  1.73 years (range 8–14 years), were recruited for this study and successfully completed the 1-year follow-up. The baseline spherical refractive error was −2.43  ±  1.27 D (range, −0.50 D to −5.50 D), and the baseline cylinder was −0.74  ±  0.43 D (range, 0.00 D to −1.75 D). During the study period, one participant (two eyes) developed grade I corneal epithelial injury due to trichiasis. After the eyelash was removed and lens wear was discontinued for 1 day, the corneal epithelium recovered. The remaining participants showed good lens fit, and no infection or other adverse events occurred during the 1-year follow-up.

### 3.1. Change in OSI, SR, and MTFcutoff

**Fig 2:**
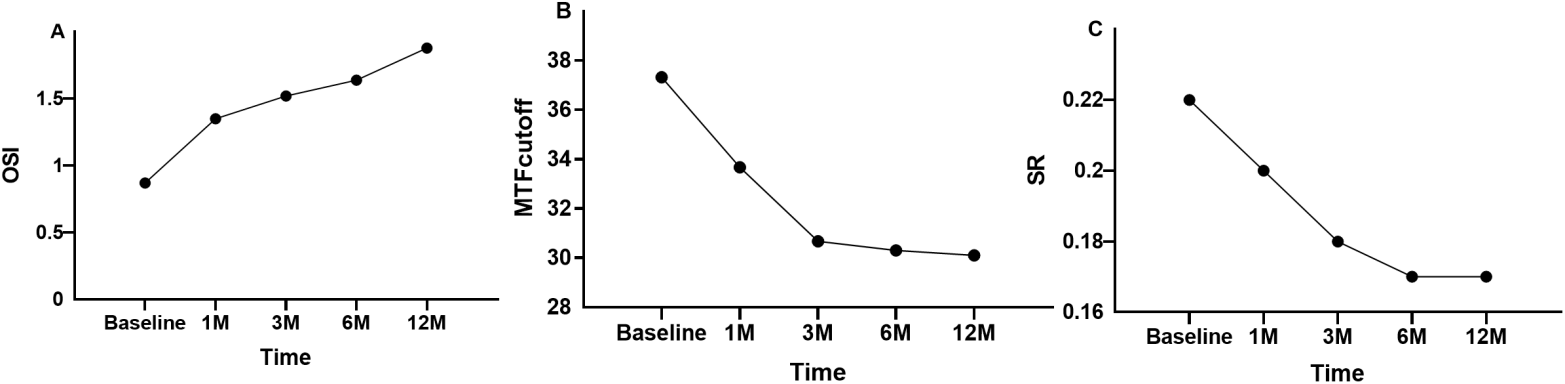
Time course of changes in objective visual quality parameters measured with OQAS II. (A) Objective scatter index (OSI); (B) Modulation transfer function cutoff frequency (MTF_cutoff_); (C) Strehl ratio (SR).

Compared with baseline, OSI increased significantly at 3, 6, and 12 months of OK lens wear (P = 0.001, P = 0.002, and P < 0.001, respectively). SR decreased significantly after 12 months of OK wear (P = 0.003). No statistically significant change was observed in MTF_cutoff_ (P > 0.05) (Table 1; Fig 2).

**Table 1:** Change in OSI, SR, and MTFcutoff during the 12-month follow-up.

	Baseline	1 month	3 months	6 months	12 months	Overall P value
OSI	0.88 ± 0.62	1.35 ± 1.07	1.54 ± 1.25a	1.70 ± 1.41a	1.88 ± 1.42a	<0.001†
SR	0.22 ± 0.08	0.20 ± 0.08	0.18 ± 0.06	0.17 ± 0.07	0.17 ± 0.05a	0.006†
MTFcutoff	37.33 ± 12.26	33.67 ± 12.39	30.74 ± 11.00	30.30 ± 12.55	30.10 ± 10.19	0.057†

Data are presented as mean ± standard deviation. MTFcutoff is expressed in cycles per degree; OSI and SR are dimensionless. OSI, objective scatter index; SR, Strehl ratio; MTF_cutoff,_ modulation transfer function cutoff frequency.

Overall P values represent comparisons across the five visits.

Superscript a indicates a significant difference versus baseline (Bonferroni-adjusted P < 0.05).

†Friedman test

### 3.2. Change in corneal wavefront aberrations

**Fig 3:**
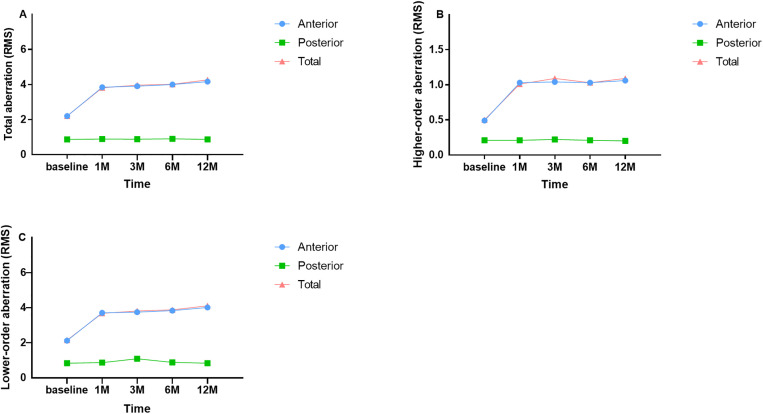
Time course of changes in corneal aberration RMS values. (A) TOA RMS; (B) HOA RMS; (C) LOA RMS.

For both the anterior cornea and total cornea, TOA RMS, HOA RMS, and LOA RMS were significantly higher at 1, 3, 6, and 12 months after OK lens wear than at baseline (all P < 0.001). No significant differences were found among the post-treatment time points (all P > 0.05), indicating that these aberration RMS values increased by 1 month after OK lens wear and remained stable thereafter. In contrast, posterior corneal TOA RMS, HOA RMS, and LOA RMS showed no significant changes throughout the follow-up period (Table 2; Fig 3).

**Table 2:** Change in corneal wavefront aberration RMS values during the 12-month follow-up.

		Baseline	1 month	3 months	6 months	12 months	Overall P value
Anterior cornea	TOA RMS	2.20 ± 1.06	3.85 ± 1.82^a^	3.91 ± 1.72^a^	4.00 ± 1.74^a^	4.17 ± 1.66^a^	<0.001†
	HOA RMS	0.49 ± 0.25	1.03 ± 0.41^a^	1.04 ± 0.37^a^	1.03 ± 0.36^a^	1.06 ± 0.43^a^	<0.001†
	LOA RMS	2.12 ± 1.03	3.71 ± 1.76^a^	3.75 ± 1.67^a^	3.84 ± 1.71^a^	4.02 ± 1.63^a^	<0.001†
Posterior cornea	TOA RMS	0.87 ± 0.16	0.89 ± 0.16	0.88 ± 0.15	0.90 ± 0.15	0.87 ± 0.16	0.84‡
	HOA RMS	0.21 ± 0.05	0.21 ± 0.04	0.22 ± 0.10	0.21 ± 0.05	0.20 ± 0.08	0.172†
	LOA RMS	0.84 ± 0.16	0.87 ± 0.16	1.09 ± 1.57	0.88 ± 0.15	0.84 ± 0.15	0.304†
Total cornea	TOA RMS	2.21 ± 1.05	3.82 ± 1.78^a^	3.97 ± 1.71^a^	4.02 ± 1.72^a^	4.27 ± 1.74^a^	<0.001†
	HOA RMS	0.50 ± 0.27	1.01 ± 0.41^a^	1.09 ± 0.49^a^	1.03 ± 0.35^a^	1.09 ± 0.44^a^	<0.001†
	LOA RMS	2.15 ± 1.03	3.68 ± 1.75^a^	3.82 ± 1.69^a^	3.88 ± 1.70^a^	4.11 ± 1.72^a^	<0.001†

Data are presented as mean ± standard deviation. All RMS values are expressed in micrometers (μm).

TOA RMS, total aberration root mean square; HOA RMS, higher-order aberration root mean square; LOA RMS, lower-order aberration root mean square. Overall P values represent comparisons across the five visits. Superscript a indicates a significant difference versus baseline (Bonferroni-adjusted P < 0.05).

†Friedman test; ‡Repeated-measures analysis of variance.

### 3.3. Change in corneal densitometry

**Fig 4:**
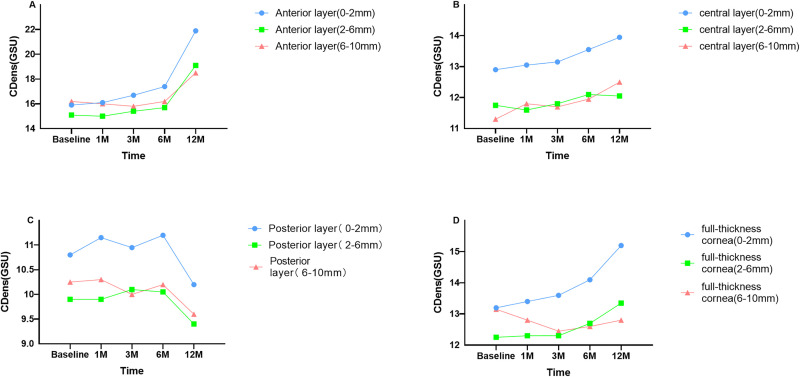
Time course of changes in corneal densitometry (CDens) in different layers and annular zones. (A) Anterior 120 μm layer (0–2 mm, 2–6 mm, 6–10 mm); (B) Central layer (0–2 mm, 2–6 mm, 6–10 mm); (C) Posterior 60 μm layer (0–2 mm, 2–6 mm, 6–10 mm); (D) Full-thickness cornea (0–2 mm, 2–6 mm, 6–10 mm).

CDens showed distinct layer- and region-specific changes during the 12-month follow-up, with the most pronounced differences observed at 12 months. In the anterior layer, CDens in the 0–6 mm region increased significantly by 6 months and remained elevated at 12 months, whereas the increase in the 6–10 mm zone was observed only at 12 months. Changes in the central layer and full-thickness cornea were mainly confined to the 0–6 mm region and were most evident at 12 months; no significant changes were observed in their 6–10 mm zones. In contrast, posterior-layer CDens decreased across all analyzed zones at 12 months. The decreases in the 2–6 mm and 6–10 mm zones were also significant compared with baseline, whereas the decrease in the 0–2 mm zone was significant compared with the 3- and 6-month visits. Detailed pairwise comparisons are presented in Table 3 and Fig 4.

**Table 3:** Change in corneal densitometry (grayscale units) across different corneal layers and annular zones during the 12-month follow-up.

		Baseline	1 month	3 months	6 months	12 months	Overall P value
Anterior layer	0–2 mm	16.00 ± 0.75	16.13 ± 0.97	16.56 ± 1.37	17.83 ± 2.51^ab^	21.05 ± 3.93^abc^	P < 0.001
	2–6 mm	14.66 ± 2.25	15.02 ± 0.88	15.16 ± 1.93	15.83 ± 1.40^ab^	18.29 ± 2.71^abc^	P < 0.001
	6–10 mm	16.88 ± 2.40	15.99 ± 2.35	16.00 ± 2.38	16.32 ± 2.51	18.30 ± 2.46^abcd^	P < 0.001
Central layer	0–2 mm	12.90 ± 0.67	12.96 ± 0.97	13.23 ± 1.49	13.50 ± 0.74	13.88 ± 1.14^abc^	P < 0.001
	2–6 mm	11.58 ± 0.59	11.67 ± 0.68	11.91 ± 0.63	12.12 ± 0.83	12.13 ± 1.33^bc^	P < 0.001
	6–10 mm	12.32 ± 1.39	12.11 ± 0.97	11.81 ± 1.45	11.91 ± 1.25	11.62 ± 1.19	0.06
Posterior layer	0–2 mm	10.83 ± 0.61	10.97 ± 0.87	10.98 ± 0.87	10.81 ± 0.81	10.13 ± 0.90^cd^	P < 0.001
	2–6 mm	10.00 ± 0.56	9.73 ± 0.62	9.95 ± 0.51	9.95 ± 0.72	9.22 ± 0.98^acd^	P < 0.001
	6–10 mm	10.16 ± 0.98	10.07 ± 0.95	10.02 ± 0.95	10.08 ± 0.87	9.68 ± 0.91^acd^	P < 0.001
Full-thickness cornea	0–2 mm	13.26 ± 0.65	13.35 ± 0.76	13.54 ± 0.79	14.12 ± 1.00^ab^	15.02 ± 1.24^abcd^	P < 0.001
	2–6 mm	12.32 ± 0.59	12.16 ± 0.70	12.32 ± 0.80	12.69 ± 0.80	13.22 ± 1.06^abc^	P < 0.001
	6–10 mm	12.73 ± 1.50	12.63 ± 1.53	12.91 ± 1.38	12.92 ± 1.37	13.21 ± 1.26	0.12

Data are presented as mean ± standard deviation in grayscale units (GSU). Overall P values were obtained using the Friedman test. Post hoc pairwise comparisons were performed using Wilcoxon signed-rank tests with Bonferroni correction. Superscript letters indicate statistically significant pairwise differences after Bonferroni correction: a = vs. baseline; b = vs. 1 month; c = vs. 3 months; d = vs. 6 months.

### 3.4. Associations of corneal densitometry with objective visual quality parameters and corneal wavefront aberration parameters

At 12 months after OK lens wear, CDens showed layer- and region-specific associations with objective visual quality parameters and corneal wavefront aberration parameters. In the anterior layer, CDens in the central and paracentral zones was weakly negatively correlated with MTFcutoff and SR and weakly positively correlated with selected TOA RMS and LOA RMS values, particularly those of the total cornea. Similar weak associations were observed in the central layer and full-thickness cornea.

**Fig 5:**
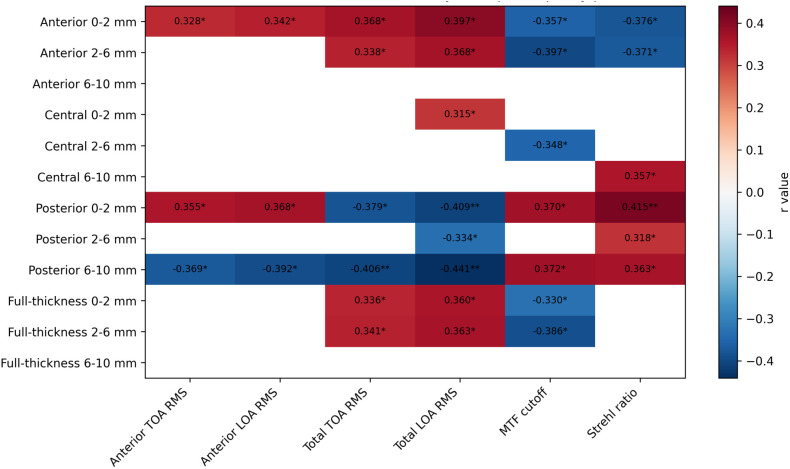
Spearman correlation heatmap between corneal densitometry (CDens) in different layers and annular zones and selected objective visual quality parameters and corneal wavefront aberration parameters at 12 months after OK lens wear. Empty cells indicate nonsignificant correlations among the variables. *P < 0.05; **P < 0.01. TOA RMS, total aberration root mean square; LOA RMS, lower-order aberration root mean square; MTF_cutoff,_ modulation transfer function cutoff frequency; SR, Strehl ratio.

In contrast, posterior-layer CDens showed an opposite pattern. In the 0–2 mm zone, posterior-layer CDens was moderately positively correlated with SR (r_s_ = 0.415, P < 0.01) and moderately negatively correlated with total corneal LOA RMS (r_s_ = −0.409, P < 0.01). In the 6–10 mm zone, posterior-layer CDens was moderately negatively correlated with total corneal TOA RMS and LOA RMS (r_s_ = −0.406 and r_s_ = −0.441, respectively; both P < 0.01). No significant correlations were found between CDens and OSI, posterior corneal aberration RMS values, or HOA RMS values (Table 4; Fig 5).

**Table 4:** Correlations of corneal densitometry with selected objective visual quality parameters and corneal wavefront aberration parameters at 12 months after OK lens wear.

Corneal densitometry	Anterior cornea		Total cornea		Objective visual quality (OQAS II)	
	TOA RMS (r_s_)	LOA RMS (r_s_)	TOA RMS (r_s_)	LOA RMS (r_s_)	MTF_cutoff_ (r_s_)	SR (r_s_)
Anterior layer						
0–2 mm	0.328*	0.342*	0.368*	0.397*	−0.357*	−0.376*
2–6 mm	0.288	0.304	0.338*	0.368*	−0.397*	−0.371*
6–10 mm	0.127	0.109	0.129	0.104	−0.112	0.005
Central layer						
0–2 mm	0.252	0.262	0.297	0.315*	−0.305	−0.240
2–6 mm	0.252	0.261	0.282	0.298	−0.348*	−0.242
6–10 mm	−0.228	−0.247	−0.259	−0.290	0.298	0.357*
Posterior layer						
0–2 mm	−0.355*	−0.368*	−0.379*	−0.409**	0.370*	0.415**
2–6 mm	−0.299	−0.312	−0.300	−0.334*	0.255	0.318*
6–10 mm	−0.369*	−0.392*	−0.406**	−0.441**	0.372*	0.363*
Full-thickness cornea						
0–2 mm	0.297	0.308	0.336*	0.360*	−0.330*	−0.307
2–6 mm	0.291	0.304	0.341*	0.363*	−0.386*	−0.301
6–10 mm	−0.081	−0.102	−0.100	−0.134	0.105	0.195

TOA RMS, total aberration root mean square; LOA RMS, lower-order aberration root mean square; MTF_cutoff,_ modulation transfer function cutoff frequency; SR, Strehl ratio.

Values are Spearman rank correlation coefficients (r_s_). Only parameters with at least one statistically significant correlation are included in the table. For the displayed parameters, both statistically significant and nonsignificant correlation coefficients are shown, with asterisks indicating statistical significance. No significant correlations were found between CDens and OSI, posterior corneal wavefront aberration RMS values, or HOA RMS values; therefore, these parameters are not shown. -1 ≤ r_s_ ≤ 1; *P < 0.05, **P < 0.01.

## 4. Discussion

Children with myopia can benefit substantially from overnight OK for myopia control [6,27]. Most studies have shown that changes in corneal surface morphology begin after approximately 1 month of OK treatment. The mid-peripheral cornea becomes steeper, whereas the central cornea becomes flatter [28, 29].

Corneal aberrations are a major component of optical aberrations in the human eye and are closely related to visual quality [30]. Batres et al. reported that the posterior corneal surface is not affected by OK lenses and that changes in corneal aberrations mainly occur on the anterior surface, which is consistent with our findings [31]. In our study, anterior corneal TOA RMS, HOA RMS, and LOA RMS increased after 1 month and then remained stable, and a similar pattern was observed for the total cornea. By inducing central flattening and mid-peripheral steepening, OK lenses alter corneal curvature, thereby generating wavefront changes. The increase in HOA RMS may reflect increased HOAs, mainly third-order aberrations such as coma and fourth-order aberrations such as spherical aberration, which are induced by corneal remodeling after OK lens wear. OK-induced increases in HOAs have been associated with glare-related visual impairment and light distortion [7, 32]. Notably, although LOA RMS might theoretically be expected to decrease as central corneal flattening reduces myopic defocus, our study found an increase in LOA RMS. This finding suggests that changes in LOA RMS may be influenced not only by the reduction in central myopic defocus caused by OK-induced corneal flattening but also by individual factors such as slight lens decentration, spatial heterogeneity of corneal epithelial remodeling, and tear-film instability [33]. Hiraoka et al. [34] also reported increased corneal irregular astigmatism despite clinically successful OK treatment, suggesting that successful correction of central myopic refractive error does not necessarily eliminate corneal optical irregularity.

After OK lens wear, intraocular scattering increased slightly, with a rise in OSI observed at 3 months. This may be related to rapid corneal remodeling, particularly thinning of the central epithelium and stroma, which may increase tissue heterogeneity and light scattering [35]. In addition to structural changes, reduced tear-film stability may also contribute to the early increase in OSI. Guo et al. [36] reported an increase in OSI after 1 month of OK wear, indicating increased intraocular scattering during the early treatment period. Reduced tear-film stability may also contribute to changes in objective visual quality after OK lens wear [37]. SR decreased only after 12 months of OK lens wear, suggesting that objective visual quality may continue to change during long-term follow-up. The MTF_cutoff_ also showed a nonsignificant downward trend from 37.33 ± 12.26 to 30.10 ± 10.19. Liu et al. reported a significant reduction in MTF_cutoff_ after 1 month of lens wear and found that this reduction was significantly associated with lens decentration [8]. The temporal trends in corneal aberration RMS values and intraocular scattering were not consistent, and corneal aberration RMS values changed earlier. After OK lens wear, factors such as lens decentration [8], tear-film stability [37], relative pupil diameter, and treatment-zone diameter may all contribute to reduced objective visual quality [38].

CDens reflects corneal transparency and can be used as an effective index for evaluating corneal health [39]. In this study, CDens showed complex and significant spatiotemporal changes across different layers after long-term OK wear. In the anterior layer, CDens increased significantly across all zones (0–2 mm, 2–6 mm, and 6–10 mm), reflecting structural remodeling and increased light scatter. This finding is consistent with Scheimpflug imaging studies showing that central flattening and mid-peripheral steepening after OK gradually affect anterior light scatter. Increased CDens in this layer may also be related to epithelial morphological changes, collagen fiber rearrangement, and mild edema [17]. In the central layer, CDens showed smaller but detectable increases, suggesting that OK-related changes may extend beyond the anterior layer. Previous work has also shown that long-term OK wear can alter corneal biomechanical properties [40], although the relationship between these biomechanical changes and CDens remains uncertain. Unlike previous studies that mainly focused on CDens changes in the anterior and mid-stroma, the present study found a significant decline in CDens within the posterior 60 μm layer across multiple central and paracentral regions after 12 months of OK lens wear. The current literature lacks systematic evaluation of long-term posterior CDens changes; most previous reports have described overall CDens increases or short-term decreases followed by rebound [17, 41]. The mechanism underlying this decline remains uncertain. Posterior corneal tissue may undergo subtle morphological or hydration-related changes in response to chronic optical and mechanical stress, although measurement artifacts related to altered light paths or local refractive-index changes cannot be excluded. Whether the observed reduction in posterior CDens reflects structural adaptation, functional alteration, or merely an imaging phenomenon requires further validation with histological and biomechanical evidence [16]. Although the CDens changes were statistically significant, their clinical relevance remains uncertain because no validated threshold for clinically meaningful Pentacam-derived CDens changes after OK lens wear has been established.

When analyzing the relationship between CDens and objective visual quality parameters, we found no significant association between CDens and OSI. This may reflect the fact that CDens represents backward scatter measured by Scheimpflug imaging, whereas OSI reflects forward scatter along the visual axis and may be more strongly influenced by tear-film instability, lens decentration, or early lenticular changes [38, 42]. In the anterior layer, increased CDens in the central and paracentral regions was negatively correlated with MTF_cutoff_ and SR, consistent with reports in healthy subjects showing that superficial scatter is an important determinant of reduced optical quality [43]. In the posterior layer, CDens was positively correlated with MTFcutoff and SR, with a moderate correlation observed between CDens in the 0–2 mm zone and SR. The mechanism underlying this finding remains uncertain. One possible explanation is that it reflects a chronic and passive form of tissue adaptation to long-term mechanical and optical stress rather than a true increase in transparency. Specifically, reductions in stromal or endothelial cell density, alterations in collagen fiber spacing or alignment, redistribution of hydration, or changes in the optical path during measurement may all contribute [43, 44]. Such changes could reduce backward light scatter measured by CDens, while not necessarily improving forward optical quality; they may even be accompanied by increased forward scatter or aberrations, ultimately leading to concurrent reductions in MTF_cutoff_ and SR. However, because the available literature on this topic remains limited, this hypothesis requires further investigation.

We also observed complex correlations between CDens and corneal aberration RMS values at 12 months. Specifically, in the anterior layer, increases in CDens in the central (0–2 mm) and paracentral (2–6 mm) zones were positively correlated with TOA RMS and LOA RMS, suggesting that superficial scatter may be closely related to lower-order optical components such as spherical error and regular astigmatism. This may result from altered epithelial thickness distribution and collagen reorganization, which increase local light scatter and reduce optical homogeneity, thereby increasing LOA RMS. In the central layer, CDens values in the paracentral zone also showed weak positive correlations with LOA RMS, suggesting that deeper stromal irregularities may influence anterior optical quality through stress transmission and collagen rearrangement [45]. By contrast, in the posterior layer, the moderate negative correlations between CDens values and both TOA RMS and LOA RMS may reflect potential alterations such as decreased stromal and endothelial cell density, changes in collagen fibril spacing or arrangement, and reduced structural support, which could make the anterior surface more susceptible to wavefront distortions [43 44]. Interestingly, although HOA RMS increased in certain regions, no significant correlations with CDens were observed. This implies that posterior or deep stromal changes may have only indirect effects on HOAs, whereas HOA RMS may be more strongly modulated by tear-film quality or lens decentration [46].

This study has several limitations. The sample size was relatively small, which prevented us from analyzing the potential effects of different diopter levels on CDens. Subjective visual quality symptoms were not assessed in this study; therefore, the relationship between objective visual quality changes and patient-reported visual experience requires further investigation. In addition, corneal microstructural and endothelial assessments, such as in vivo confocal microscopy and specular microscopy, were not performed. The lack of these biological and structural data, together with the absence of follow-up after treatment discontinuation, limits the mechanistic interpretation of posterior CDens changes. Nevertheless, to our knowledge, few studies have reported the correlation between objective visual quality and CDens after long-term OK treatment. Further studies are needed to verify these results.

## 5. Conclusion

Consistent with previous studies, 1 year of OK lens wear was associated with persistent increases in anterior and total corneal aberration RMS values and changes in selected objective visual quality parameters in adolescents with myopia. Corneal densitometry showed distinct layer- and region-specific changes, with anterior densitometry generally increasing and posterior densitometry showing a decreasing trend. Importantly, the correlations between posterior corneal densitometry and objective visual quality parameters suggest that posterior corneal densitometry may provide complementary information for evaluating long-term optical quality in eyes treated with OK lenses. However, the clinical significance of these findings remains uncertain and warrants confirmation in larger studies.

## Supporting information

S1 Data Raw data used for the analyses of objective visual quality, corneal wavefront aberrations, and corneal densitometry during the 12-month follow-up. (XLSX)
